# “All you Gain is Pain and Sorrow”: Facilitators and Barriers to the Prevention of Female Genital Mutilation in High-income Countries

**DOI:** 10.1177/15248380241229744

**Published:** 2024-02-16

**Authors:** Fatima Younas, Leslie Morrison Gutman

**Affiliations:** 1 University College London, UK

**Keywords:** female genital mutilation, barriers, facilitators, high-income countries

## Abstract

**Background:**

Female genital mutilation (FGM) is a harmful practice that has long-lasting negative impacts on the physical and psychological health of victims. Deemed a global concern, this practice persists in high-income countries (HIC) among certain migrant communities. Given the deleterious effects of the practice, we conducted an updated systematic review of the facilitators and barriers associated with the prevention of FGM in HIC.

**Method:**

A systematic review of published qualitative studies of FGM in HIC was conducted from 2012 to 2022. The search resulted in 276 studies. Of these, the majority were from low- and middle-income countries (LMIC) and excluded. A total of 14 studies were deemed fit for inclusion and none were excluded during quality appraisal. Relevant data were extracted from the studies and thematically analyzed to identify prevalent themes.

**Results:**

A total of 12 themes were identified and the majority reflected barriers to the prevention of FGM including beliefs about female virtue, beliefs about social sanctions, and the preservation of culture, among others. Facilitators to the prevention of FGM were fewer and included memory and trauma from experiencing FGM, knowledge and awareness of the female anatomy, and legislative protection from FGM due to migration. A few themes, such as religious beliefs, acted as both facilitators and barriers.

**Conclusion:**

Findings highlight the importance of shared cultural and social threads among FGM practicing communities in HIC. Interventions can use these findings to guide the development of sociocultural strategies centered on community-level prevention and reduction of FGM in HIC.

According to the World Health Organization (WHO, 2019), female genital mutilation (FGM) is a traditional and harmful practice which involves complete or partial removal of the female genitalia (external) or injury to the female genital organs for non-medical purposes. FGM is a global concern and is practiced in over 30 countries across North and Sub-Saharan Africa, in parts of the Middle East, and Asia (UNICEF, 2010). While the prevalence of FGM is difficult to ascertain due to under-reporting, a recent systematic review of studies across 30 countries from 2009 to 2022 estimates that 100 million girls and women globally have experienced FGM (Farouki et al., 2022). Prior research has established the negative consequences of FGM including immediate health risks such as severe pain and shock (Kontoyannis & Katsetos, 2010), hemorrhage and infections (Berg & Underland, 2013), and psychological consequences such as post-traumatic stress disorder (Behrendt & Moritz, 2005). Long-term health risks include chronic pain (Hadid & Dahan, 2015), infertility (Reisel & Creighton, 2014), birth complications (Morison et al., 2001), and sexual dysfunction (Alsibiani & Rouzi, 2010), among others.

Due to a rise in migration, many high-income countries (HIC) are reporting increasing numbers of women and girls who have undergone or are at risk of FGM (Barrett et al., 2020). Estimates of prevalence in 2015 from European countries show that nearly half a million women have undergone FGM (Barrett et al., 2020). Based on figures from 2012 and 2015, it was estimated that 137,000 women and girls had FGM in the UK and USA, and a further 507,000 were at risk (Goldberg et al., 2015; Mcfarlane & Dorkenoo, 2015). For some migrants, research suggests that moving to HIC strengthened their beliefs regarding the perpetuation of FGM as an “identity marker” (Alhassan et al., 2016). Despite interventions, criminalization, and campaigns to end FGM within HIC, the practice continues (Hemmings, 2011; Nijboer et al., 2010).

As FGM is performed on an individual within a family and is supported by cultural, social, and community traditions, it is important to identify the influences on this behavior in the context in which it occurs for prevention purposes (Barrett et al., 2020). Our systematic review presents an update to Berg and Denison’s (2013) systematic review, as the prior review was conducted nearly a decade ago and evidence in the field is continually evolving. With greater awareness of FGM, along with the increase in interventions targeting its prevention in HIC (Njue et al., 2019), the identification of new studies in an updated systematic review can help to refine or even change previous conclusions and simultaneously enable stakeholders to keep abreast of the latest evidence in the field (Garner et al., 2016). While Berg and Denison’s (2013) review included quantitative studies, ours is a qualitative synthesis of the facilitators and barriers to the prevention of FGM in HIC. Qualitative evidence can provide useful and detailed insight into how and why FGM occurs in HIC (Williams et al., 2018).

## Research Background

The majority of studies examining the facilitators and barriers to the prevention of FGM are based in low-income countries (LIC) countries where this practice is common. This research emphasizes the cultural, social, and religious influences on FGM. For example, research conducted in Sudan identified influences such as adhering to traditions and social norms, transition to womanhood, and religious motivations to continue FGM, among others (Gruenbaum, 2005, 2006; UNICEF, 2010). Relatedly, Bosire (2013) describes the Bondo society in Sierra Leone in which politicians reinforce FGM as part of an initiation to womanhood (named sisterhood) and women who have had FGM wear white headscarves as a mark of their transition. The symbolic ritual represents cultural identification and enables cohesiveness among members, leaving little room to question or abandon FGM (The New Humanitarian, 2005). Social sanctions are also evident for those who defy this practice. Burrage (2015), for instance, found that girls from communities which traditionally practice FGM but reject it, are considered promiscuous and socially excluded. With respect to religion, there are certain communities in the Christian (e.g., Coptic Christians in Egypt and Sudan), Jewish (Falashas in Ethiopia), and Muslim faiths which base the practice of FGM on religious grounds (Aldeeb Abu-Sahlieh, 1995; El-Damanhoury, 2013). Cohen (2005) argues that it is not religion which is the reinforcer but the coincidental occurrence of religious communities practicing FGM in geographical areas where the prevalence is high.

While these studies highlight the factors that contribute to the continuation of FGM, fewer studies have investigated the influences on its prevention. In exception, a prior systematic review included studies from 11 LIC and four HIC and examined the role of men in rejecting FGM (Varol et al., 2015). The review found education and knowledge of health complications of FGM among men were enablers to the abandonment of FGM, while social obligation was a barrier to its prevention. Berg and Denison’s (2013) systematic review found several cultural influences acting as barriers to prevention, including marriageability, religion, sexual morality, honor, and cultural tradition. Among facilitators, they found legislation, religion, and health consequences helpful in abandoning the practice of FGM in HIC. Other existing systematic reviews of FGM have focused primarily on quantitative evidence which is unable to provide a nuanced picture of the barriers and facilitators to this practice (e.g., Evans et al., 2019). The scant qualitative systematic reviews tend to emphasize health care provision and access for FGM victims. For instance, one such review explored the barriers and facilitators to health care provision for survivors from the perspective of health care practitioners (Catrin, 2020). Another systematic review focused on short-term interventions for FGM in HIC, finding no long-term improvement and emphasizing the paucity of interventions that effectively address the cultural and social processes underlying the perpetuation of this practice (Njue et al., 2019).

Gaps in knowledge include a need for “further exploration of the determinants of the practice and of resistance to its abandonment. . .” along with “contextual analysis of the mechanisms of the practice of FGM” (Andro & Lesclingand, 2016, p. 271). Due to the nature of FGM as part of a complex socio-cultural system, a greater focus should be placed on the underlying motivations of the behavior to bring about sustained change (EIGE, 2013). While many of the socio-cultural influences are transferable from LMIC to HIC, prior studies have noted differences in FGM prevalence among rural and urban areas (Andro & Lesclingand, 2016), education levels (Afifi, 2009), and the presence of laws supporting the abandonment of FGM (Leye & Deblonde, 2004). Some scholarly discourse about FGM in HIC has been criticized for not being applicable to communities that perpetuate this practice (Barry, 2015). Criticisms have also been levied against certain HIC governmental approaches and safeguarding practices to tackling FGM citing that these efforts are based on stereotypes, are heavy-handed and discriminatory, lack evidence, and are not culturally competent (Karlsen et al., 2020). Njue et al.’s (2019) systematic review of interventions for the prevention of FGM in HIC found limited evidence of effectiveness and noted a focus on legislative action and health persuasion by interventions which “do not appear to be justified by a strategic approach that is informed by baseline research” (Njue et al., 2019, p. 16). Furthermore, Sheerin et al.’s (2023) systematic review found that HIC health workers had limited knowledge about FGM which affected their quality of care. Similarly, studies have found HIC health workers’ desire to acquire more training on FGM including knowledge about legislation and cultural influences (Lane et al., 2019; Turkmani et al., 2018). Hence, further understanding is needed about the motivating influences underlying both the ceasing and continuance of FGM in the context of HIC to clarify and update knowledge in the field, aid health workers to identify and care for FGM victims, and for intervention practitioners to develop effective strategies.

To this end, our study systematically reviews and synthesizes evidence on FGM through elucidating underlying motivations that either perpetuate the practice (barriers) or help to stop it (facilitators) in HIC. An updated qualitative systematic review of the facilitators and barriers to the prevention of FGM in HIC can inform the development and tailoring of preventive interventions specifically for HIC and guide further research to alleviate this harmful practice.

## Method

### Systematic Review: Inclusion Criteria and Search Strategy

The inclusion criteria, as displayed in Table 1, consisted of empirical, qualitative research published in a journal from 2012 to 2022, which focused on barriers and facilitators of FGM prevention in HIC. Quantitative studies and those focusing on LIC were excluded along with editorials and opinion pieces.

**Table 1.** Inclusion Criteria for Study Selection.

Domain	Inclusion Criteria
Publication	Empirical, published journal articles
Study year	2012–2022
Participants	FGM survivors, those at risk of FGM, health care professionals related to FGM, and other stakeholders, including families, communities, social workers, and intervention practitioners for FGM
Focus of study and methods	Qualitative studies that include facilitators and/or barriers to the practice of FGM in HIC
Excluded Studies	Opinion pieces, editorials, quantitative studies, studies conducted in LIC

*Note.* FGM = Female genital mutilation; HIC = High-income countries; LIC = Low-income countries.

Databases searched included PsycINFO, Web of Science, and SCOPUS. Table 2 shows the search terms and results from each database.

**Table 2.** Results from Database Searches.

PsycINFO	Filters	Results
(“Female genital mutilation” OR FGM OR “female genital circumcision” OR “female genital cutting”) AND (barrier OR obstacle OR hurdle OR facilitator OR perpetuate OR driver OR motivation)	Advanced search—filters: journal article; year 2012–2022; filter by keyword—female genitalia	20
Web of Science		
(“Female genital mutilation” OR FGM OR “female genital circumcision” OR “female genital cutting”) AND (barrier OR obstacle OR hurdle OR facilitator OR perpetuate OR driver OR motivation)	Advanced Search - Filters: Journal article; Year—2012–2022; further refined by excluding journals (e.g., mechanical/engineering journals)	61
SCOPUS		
(“Female genital mutilation” OR FGM OR “female genital circumcision” OR “female genital cutting” OR “FGM/C”) AND (barrier OR obstacle OR hurdle OR facilitator OR perpetuate OR driver OR motivation OR influence)	Advanced Search - Filters: Journal article; subject—medicine, social science, psychology, nursing; filter by keyword—“Female genital mutilation”	195
Total		**276**

A search of the databases was conducted in December 2022, and a total of 276 studies were found. EPPI reviewer (Thomas et al., 2010), a software tool for research synthesis, found 37 duplicate references, and these were deleted, leaving a total of 239 studies. All references were stored in EPPI Reviewer 4 (Thomas et al., 2010) and screening, data extraction, and quality assessment were undertaken using this software.

### Screening

**Figure 1.**
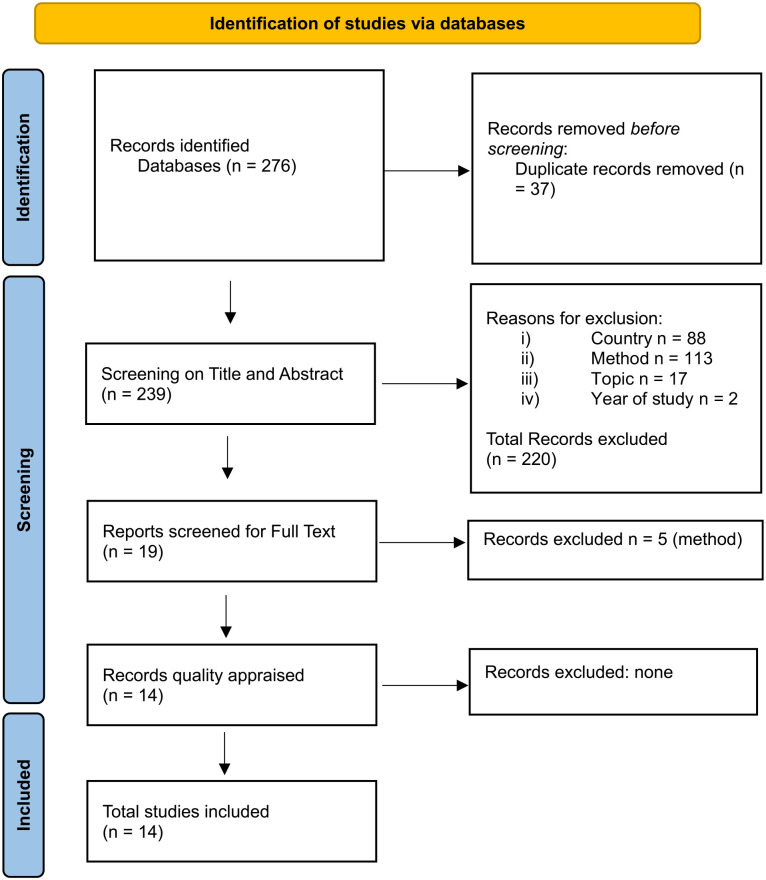
Preferred Reporting Items for Systematic Reviews and Meta-Analysis flow chart.

Figure 1 displays the Preferred Reporting Items for Systematic Reviews and Meta-Analysis (PRISMA; Page et al., 2021). From the 239 studies, screening on title and abstract resulted in the inclusion of 19 studies and the exclusion of 257 studies. To establish reliability, the second author screened 10% of the 220 studies for the title and abstract, and inter-rater reliability was established at 100%, with no differences in decisions of inclusion or exclusion noted. The majority of the studies were excluded as they were not conducted in HIC, and the remaining were excluded because of the method, relevance of topic and/or year of the study. The remaining 19 studies were retrieved from electronic sources, and full-text screening was conducted, resulting in the exclusion of five additional studies due to the study methods. The second author conducted full-text screening on 10% of the 14 studies, and inter-rater reliability was established at 87%. Discrepancies were resolved between the authors through discussion until a 100% agreement was reached. A final fourteen studies were included and appraised for quality.

### Quality Assessment

Quality appraisal of the 14 included studies was done using the Critical Appraisal Skills Program (CASP, 2022) qualitative studies checklist (see Supplemental File 2). The CASP checklist considers the validity of the results and asks questions concerning the clarity of the aims of the study, appropriateness of methods, research design, and recruitment strategies, as well as data collection. It also asks about the study authors’ consideration of relevant ethical issues and whether rigor is evident in the data analysis. Finally, quality is assessed through questions concerning the value of the study and the applicability of the findings to relevant and local populations.

A modified version of the Grading of Recommendations, Assessment, Development and Evaluation (GRADE) approach (Ryan & Hill, 2016) was applied to rank the studies. Based on this approach, qualitative studies are initially given a ranking of “low” and moved up to a ranking of “medium” or “high” or moved down to a ranking of “very low” based on the assessment criteria. Studies were judged on four broad criteria from the CASP checklist: rigor in data collection, rigor in analysis, validity of results, and applicability of findings. Studies marked “very low” were considered fit for exclusion while those marked high, moderate, or low were included.

### Data Extraction

A data extraction form was devised, which included administrative details of the study and general study characteristics, including aims of study, theoretical underpinnings, sample, method, and data collection and analysis (See Supplemental File 1). Finally, findings of the study associated with the motivations for perpetuating FGM and those influences that help prevent FGM were recorded. The data were extracted from included studies along with relevant quotes for the purposes of data analysis.

### Data Analysis

Following quality appraisal and the extraction of relevant data from the included studies, an inductive thematic analysis process (Braun and Clarke, 2006) was utilized. A thorough reading and re-reading of the extracted data was done for familiarity. Emergent themes from the data were identified and labeled, and patterns were noted across studies. Themes were classified according to whether they represented a barrier or facilitator to FGM prevention in HIC. These themes were supported with direct quotes from participants from the included studies. Reliability between the study authors in identifying and classifying themes was initially established at 75%. Discrepancies were discussed and changes were made accordingly until 100% agreement was obtained.

## Findings

### Quality Appraisal

Table 3 displays the quality appraisal rankings for each study based on the four broad assessment criteria. Three studies were ranked as moderate quality, while eleven studies were ranked as high quality. Studies ranked high quality had appropriate data collection and data analysis, results appeared valid, and findings were applicable to the represented population. For studies marked moderate, there were some limitations noted in the applicability of findings and a lack of rigor in the data collection and/or data analysis methods. No study was ranked “very low,” and thus, none were excluded based on quality criteria.

**Table 3.** Quality Appraisal Results.

Study	Rigor in Data collection	Rigor in Data analysis	Validity of results	Applicability of findings	Final Grade
Abgoli, Richard et al. (2020)	High	High	Moderate	Moderate	Moderate
Abgoli, Botbol et al. (2020)	Moderate	High	High	High	High
Alhassan et al. (2016)	High	Moderate	High	High	High
Connelly et al. (2018)	Moderate	High	High	Moderate	Moderate
Glover et al. (2017)	High	High	High	High	High
Isman et al. (2013)	High	High	High	High	High
Johansen (2017)	High	High	High	Moderate	High
Johansen and Ahmed (2021)	Moderate	Moderate	Moderate	Moderate	Moderate
Johnson-Agbakwu et al. (2014)	Moderate	High	High	High	High
Jordal et al. (2019)	High	High	High	High	High
Koukoui and Guzder (2017)	High	High	High	High	High
Mastrullo et al. (2016)	High	High	High	Moderate	High
Ruiz et al. (2017)	High	High	High	High	High
Strid and Axelsson (2020)	Moderate	High	High	High	High

### Study Characteristics

Fourteen qualitative studies fit the inclusion criteria. Table 4 depicts the characteristics of the included studies. The total number of participants from all the studies equaled 560. Of the 14 studies, 59% had both male and female participants, 23% had only male participants, and 18% had only female participants. Age of the participants ranged from 18 to 65 years. Thirteen studies included participants who originated from a country where FGM was practiced (e.g., Sudan and Somalia) and one study (Connelly et al., 2018) included stakeholders (e.g., academics, police officers, and NGO workers) from various HIC (e.g., Belgium and Spain). Six of the 14 studies used only interviews for data collection and one study collected data through focus groups. The remaining seven studies used more than one data collection method including interviews, focus groups, participant observations, and informal chats.

**Table 4.** Characteristics of Included Studies.

Study	Aims	Country	Study Sample	Sample Age (years)	Data Collection Method
Abgoli, Richard et al. (2020)	This study examined what makes individual migrant women in Belgium (originating from an FGM practicing culture) change their attitudes toward the practice and speak out against it.	Belgium	15 women, living in Belgium (for one year or more) had experienced FGM but were now against the practice. All came from Sub-Saharan Africa.	23–50	Interviews
Abgoli, Botbol et al. (2020)	This study aimed to first, describe the aftermath of disempowerment caused by FGM of the migrant women in relation to the central human capabilities, and second, to discuss the process of empowerment for these women.	Belgium	Nine migrant women seeking asylum in Belgium who had undergone FGM	18–50	Interviews
Alhassan et al., (2016)	This study explored the dynamic nature of belief systems and enforcement mechanisms that perpetuate FGM among three African migrant communities in the EU.	Italy, Portugal, and Spain	275 participants (male and female) were aged 18 years and above; and originated from the designated FGM practicing country (e.g., Eritrea/Ethiopia, Guinea Bissau, and Senegal/Gambia).	18–65	Interviews, focus groups
Connelly et al. (2018)	This study explored the role of potentially affected communities to address FGM in Europe, examining current practices, promising interventions, and remaining gaps.	Spain, France, Netherlands, England, Belgium, Ireland, Scotland	54 total participants—academics, police officers, NGO workers, policy makers, and community activists; with 36 for group interviews and 18 for individual interviews	Not stated	Interviews, focus groups
Glover et al. (2017)	This study attempted to gain an understanding of women’s experiences of FGM to develop an evidence-based holistic conceptual framework.	UK	20 female participants over the age of 18, living in the UK who had experienced FGM as a child in their country of origin	Mean 38 years	Interviews
Isman et al. (2013)	This study explored how women from parts of the world where female genital mutilation (FGM) is normative, perceive and experience FGM after immigrating to Sweden.	Sweden	Eight women—with origins from a country where FGM is a normative practice and a resident in Sweden.	19–46	Interviews
Johansen (2017)	This study aimed to explore the dynamics of change in meaning-making about female genital cutting among migrants from Somalia and Sudan residing in Norway.	Norway	36 Sudanese and Somali-originating men and women who migrated to Norway. All women experienced FGM.	18–65 with the majority 30–40 years	Interviews, informal chats, participant observation
Johansen and Ahmed (2021)	The aim of this study was to explore migrant Somali and Sudanese women’s reflections and decision-making regarding female genital cutting in a transnational context.	Norway	23 women—15 Somali and 8 Sudanese women residing in Norway (21 subjected to FGM)	Not stated	Interviews, observations
Johnson-Agbakwu et al. (2014)	This study examined the perspectives of Somali men toward FGM in one refugee community in the USA.	USA	Eight Somali-born male refugees over the age of 18, residing in Arizona. Participants in the individual interviews spent 15 years displaced as refugees in the neighboring countries prior to resettlement in the USA.	18–72	Interviews, focus groups
Jordal et al. (2019)	This study explored women’s perspective of FGM.	Sweden	17 FGM-affected women	19–56	Interviews
Koukoui and Guzder (2017)	The aim of this study was to shed light on mothers’ perception of the meaning and cultural significance of the practice and to gain insight into their mothering experience of “uncut” girls.	Canada	Seven women who lived in Montreal, Canada. Selection criteria were for women to have undergone FGM/C and to have at least one daughter who did not undergo the practice	28–62	Interviews
Mastrullo et al. (2016)	This study aimed to investigate the attitudes, knowledge, and beliefs regarding female genital mutilation/cutting (FGM) of immigrant men.	Italy	50 men, originally from 6 different African countries, currently residing in Italy	Mean 36 years	Focus groups
Ruiz et al. (2017)	The aim of this study was to detect the weak points and false premises underlying male justification of FGM	Spain and Morocco	25 men—originating from countries where FGM is performed, familiar with FGM, personal contact with women who experienced FGM, lived at least 18 years of their life in their country of origin	20–53	Individual interviews, focus groups
Strid and Axelsson (2020)	This study explored the roles of men in the continuation/abandonment of FGM in a migrant minority community in Sweden.	Sweden	13 first and second-generation male minority migrants in Sweden	27–63	Interviews, focus groups

*Note.* FGM = Female genital mutilation.

### Themes

Twelve inductive themes were identified, and these are shown in Table 5, along with their frequency and classification as barriers and/or facilitators. There were some overlaps in the identification of the facilitators and barriers across the themes; facilitators to FGM prevention in HIC were identified in six themes, while barriers that enabled FGM were identified in nine themes.

**Table 5.** Facilitators and Barriers of FGM Prevention and Frequency.

Themes	Facilitators/Barriers	No. of Studies (*n* = 14)
Beliefs about female virtue	Barrier	10
Preserving culture and influence of community elders	Barrier	7
Protection from FGM based on migration to HIC	Facilitator	7
Beliefs about social sanctions	Barrier	6
Knowledge and awareness	Barrier/Facilitator	6
Maintaining intergenerational traditions	Barrier	5
Religious beliefs	Barrier/Facilitator	5
Breaking the intergenerational cycle	Facilitator	4
FGM associated with cleanliness	Barrier	4
Parental influence	Barrier/Facilitator	3
Culture of silence	Barrier	3
Memory of trauma/pain	Facilitator	2

*Note.* FGM = Female Genital Mutilation; HIC = High Income Countries.

#### Beliefs About Female Virtue—Barrier

This was the most prevalent theme. The theme emphasized the belief that practicing FGM would ensure a woman remains a virgin before marriage, for instance, in one study a male participant noted that “. . .if the girls are not and they will lose their virginity. And that’s a shame” (Koukoi & Guzder, 2017). There was also a belief that FGM promotes chastity and faithfulness to the husband after marriage. “When I am ready for marriage, I will like to go back to my village and find a wife. Here it is difficult to find a good wife. They are not cut so they are not pure [. . .] Women who are cut are faithful to their husbands” (Alhassan et al., 2016). The underlying mechanism of FGM to ensure female virtue by reducing sexual pleasure and desire in women was also emphasized. “It is to reduce the pleasure in women. It is done to reduce the need in women if the man has to travel to earn a living for the family. I think this is the main reason” (Ruiz et al., 2017). A female participant in one study stated, “We do it to reduce the sexual obsession of our girls, and not to maltreat them. You know, we women are very weak so if we don’t protect our daughters, they cannot resist. . . they will become prostitutes” (Alhassan et al., 2016).

#### Preserving Culture and Influence of Community Elders—Barrier

Another prevalent theme focused on culture and its preservation through perpetuating the traditional practice of FGM. “I think you preserve it (the tradition) because of culture, to have something cultural” (Isman et al., 2013). This was intertwined with the respect and influence of community elders who encouraged the practice as a means of maintaining a connection with their cultural roots. “The elders in this community are well respected. They are those who protect our culture. They have a lot of experience, and we respect them. So, when they recommend that you subject your daughter to FGM, you have to respect that” (Alhassan et al., 2016).

#### Protection from FGM Based on Migration to HIC—Facilitator

This theme encapsulated the facilitative influence of migrating to a host country. Participants felt protected from FGM by HIC laws. One participant noted that “. . .women felt that they were in a better environment for their daughters, an environment where the practice of FGM is frowned upon and illegal and where they are protected from this practice” (Abgoli, Botbol et al., 2020). While another stated, “. . .living in Sweden had significantly influenced how the women saw FGC [female genital cutting], as they were exposed to anti-FGC messages and new ideals regarding body image and female sexuality there” (Jordal et al., 2019). Another study noted that moving to HIC enabled abandonment of the practice, “. . .she had no intention to cut her daughter, partly because she knew it to be illegal in Norway” (Johansen & Ahmed, 2021). There was also an element of social influence noted upon migration which facilitated the prevention of FGM. One female participant stated, “when you come here, you discover that not all women are like you” (Abgoli, Richard et al., 2020).

#### Beliefs About Social Sanctions—Barrier

This theme centered on participants’ beliefs that they would be penalized by other community members if they did not practice FGM. “The thing is you don’t want your daughter to be called solima (rude, uncivilized) when she goes home. . .some people do it [FGM] because of that” (Alhassan et al., 2016). Similarly, in one study a participant observed that ‘uncut’ daughters and their family are typically shunned from the community and are prevented from partaking in traditional ceremonies and specific group gatherings” (Koukoi & Guzder, 2017). Others spoke about their own experiences of facing social sanctions as a result of discontinuing the practice. One female participant who was ‘uncut’ spoke about her experience and stated, “Somali families cross to the other side of the street when they meet me. I think they are scared I will have a bad influence on their daughters” (Johansen & Ahmed, 2021). For instance, there was also a fear expressed by some that without FGM, their daughters may not find a husband. “Mothers are also afraid that their daughters might not be married if they are not circumcised” (Johnson-Agbakwu et al., 2014). Another participant noted, “I have undergone female circumcision and I asked for it because I was fed up with being excluded from playing with my friends” (Abgoli, Richard et al., 2020).

#### Knowledge and Awareness—Facilitator/Barrier

Knowledge and awareness acted as both a facilitator and a barrier to FGM prevention. Studies reflecting this theme had a majority of female participants who experienced FGM and upon migration to HIC became more aware of laws protecting them, which was a facilitator. Expressing a lack of knowledge, however, was a barrier to prevention. One female stated, “I had no idea, I didn’t know about the law. I talk to others now about the law and that it’s forbidden in Sweden, but there are many who don’t know” (Isman et al., 2013). Another participant expressed a lack of prior knowledge about the female anatomy and her feelings upon becoming more knowledgeable. “But during my studies, I realized some things and it was a shock . . . The first time I saw the genital organ of a woman, I said ah . . . so I lost this part of me in the excision” (Abgoli, Richard et al., 2020). The acquired knowledge was considered a facilitator.

#### Maintaining Intergenerational Traditions—Barrier

An intergenerational transmission of FGM and its value in its maintenance was noted in five studies. Participants wished to maintain tradition through the generations, noting that, “Our ancestors did it and they expect us to do it in their absence” (Alhassan et al., 2016). The value of passing on the practice to future generations as a custom was noted by another participant, “We were told that a girl has to go through that, and we should pass it on to the next generation. They have gone through it, so we have to go through it too. That’s how it is, it’s a custom to be respected. . .” (Abgoli, Richard et al., 2020).

#### Religious Beliefs—Facilitator/Barrier

This theme acted as both a facilitator and a barrier to the prevention of FGM. One participant noted the influence of religion on facilitating a change in perspective, which enabled stopping the practice. “We went to an Imam and thought that they could help us with this. . .and since it’s not in the religion, it changed my mother’s view on all of it” (Isman et al., 2013). Another participant highlighted the role of social influences, which acted as a barrier to FGM prevention, and stated, “In my country. everyone does it [FGM]. . . we all do it especially in the villages. . .once a baby is born whether boy or girl, the religion expects that they are circumcised” (Alhassan et al., 2016). There was also an emphasis on FGM and a belief that its practice would be rewarded in religion as one participant noted, “Most . . .perceived that it [FGM] was a good deed that would earn them divine reward” (Alhassan et al., 2016).

#### Breaking the Intergenerational Cycle—Facilitator

Four of the included studies had participants who were motivated to break the cycle of FGM across generations. Opposition to the transmission of the practice arose from underlying emotions and pain associated with experiencing FGM, which acted as motivators to break the cycle. “I was destroyed by my mother and my grandmother. I can say that since they have done something horrible to me . . . I love them but when my in-laws wanted to excise my daughter, as was usual. But I opposed” (Abgoli, Richard et al., 2020). Another participant stated, “I regret it [undergoing FGM] so much! So much! If someone came to me now telling me ‘I’ll give you billions of dollars if you circumcise your daughter’, I can’t! I can’t! I can’t because of the pain that I know today” (Koukoi & Guzder, 2017).

#### FGM Associated with Cleanliness—Barrier

Beliefs about FGM enabling female cleanliness and hygiene were noted in this theme. One participant noted that if FGM is not practiced then, “. . .the clitoris will grow and they will have many infections” (Ruiz et al., 2017). One parent stated, “if your daughter isn’t circumcised, she isn’t clean” (Isman et al., 2013). In another study, a participant correlated health and cleanliness stating, “For health’s sake, they [those who have FGM] are cleaner” (Glover et al., 2017).

#### Parental Influence—Facilitator/Barrier

Parental influence acted as both a facilitator and a barrier. In the three studies which highlighted this theme, only one participant advocated stopping the practice stating, “but as a parent you’d never abandon your children, I think. So, you should say to the girls, be strong in your opinions and your parents will never abandon you, don’t do this. Don’t listen to old tradition” (Isman et al., 2013). Parental influence as a barrier was represented in the remaining two studies with one participant noting a stronger influence from their father, “dad was stronger than Mum, he knew I need FGM or wouldn’t get married. He didn’t need my mum’s permission” (Glover et al., 2017). While in Strid and Axelsson (2020), a female participant stated that the mother plays a bigger role in FGM decision-making, “In Somalia, our dad has never told us or talked about it. It is the mothers that take care of the girls, if they shall be cut or not. The dad has nothing to do with it”.

#### Culture of Silence—Barrier

This theme represented a barrier to FGM prevention. It captured participants’ views on not being able to talk about FGM and any related issues. A general taboo around the subject was noted among participants, especially with reporting of FGM. One participant, speaking from the perspective of FGM victims, noted, “. . .the likelihood that a survivor would need to testify against her relatives holds many back from reporting FGM” (Mastrullo et al., 2016). While another spoke of the general environment which discourages speaking about FGM, “We were forbidden to tell others what had happened. . . Nobody spoke about how it happened” (Abgoli, Richard et al., 2020).

#### Memory of Trauma/Pain—Facilitator

The memory of experiencing FGM and the associated pain and trauma acted as a motivator for victims to stop the perpetuation of the practice. This theme was noted in two studies and emphasized the trauma endured by victims as one participant stated, “it must be said that this operation is very traumatic. We only perpetuate the tradition of our ancestors. All you gain is pain and sorrow” (Abgoli, Richard et al., 2020). Another spoke about the harmful and persistent impact of FGM throughout life, “But then what we do not understand is how much it hurts. . .it’s horrible, and it follows you everywhere. . .in adulthood, in your teenage years when menstruating, when you get married, when you have sex with your husband, if you give birth, if you go through a caesarean . . . you see? The pain follows you everywhere, and it’s horrible” (Abgoli, Richard et al., 2020).

## Discussion

This research systematically reviewed and synthesized qualitative evidence from 14 studies on the facilitators and barriers to FGM prevention in HIC from 2012 to 2022 as an update to Berg and Denison’s (2013) systematic review. Many of our review’s findings, especially barriers to the prevention of FGM, are reflective of Berg and Denison’s (2013) review. These include barriers to prevention such as religious influence and the role of sociocultural traditions and beliefs as well as a few facilitative influences including HIC legislation and the influence of religion. Our review, however, uncovered a few themes that are unique and add further knowledge to the field. Among these, the role of parental influence and the continuance/discontinuance of intergenerational traditions acted as both facilitators and barriers to FGM prevention. We also found that religion acted as both a barrier and facilitator to FGM prevention in HIC. Our review recommends potential strategies for interventions which can aid the prevention of FGM in HIC.

From our review’s findings, some overlaps were noted in classifying the themes, and three themes acted as both facilitators and barriers. From the remaining nine themes, six were classified as barriers and three as facilitators to preventing FGM in HIC. Facilitators to the prevention of FGM included memory of the trauma and pain, knowledge and awareness, and breaking the cycle of intergenerational transmission. Barriers included a culture of silence, beliefs about female virtue, and social sanctions. Themes that acted as both barriers and facilitators included religious beliefs, parental influence, and knowledge and awareness. Prevalent and key themes found in our review are discussed in this section.

In line with prior research (e.g., UNICEF, 2010; WHO, 2011), a key barrier to the prevention of FGM relates to core cultural values especially pertaining to women’s virtue. The findings from our systematic review highlight this barrier as rooted in the minds of mostly male participants, focusing on women’s honor, faithfulness, prevention of promiscuity, and marriageability. Mackie (2000) theorizes that the emphasis on female virtue among FGM-practicing communities initially came from a focus on male virility and establishing paternity. Later, the practice normalized into an inference that women must be ‘wanton’ to require control of their excess sexual urges through FGM. Mackie (2000) refers to this as a ‘morality code’ prevalent in some Sudanese and Somalian communities. Our findings further highlight this notion that female sexual urges require control. While the majority of the participants in our included studies reflecting this theme were male, prior evidence suggests that this belief was also mirrored among females. For instance, one study explored 95 Nigerian mothers’ views on FGM and found that over half of the mothers reported awareness of health risks from FGM, yet nearly half of them also reported a fear of their daughters becoming promiscuous without FGM (Ahanou & Victor, 2014). Research shows that beliefs about female virtue, however, are not rooted in reality. For instance, Barry’s (2015) study in Guinea showed that adolescent girls who had experienced FGM were sexually active before marriage, emphasizing the misguided notions prevalent in the belief structure. A systematic review of interventions in HIC for FGM found that many of the interventions focus on legislative action and health persuasion, which have limited effectiveness and many are not supported by research evidence (Njue et al., 2019). A focus on some of the nuanced cultural and social motivations that underlie the practice of FGM, such as targeting misguided beliefs, may be effective in FGM prevention. Prior research has found that community engagement programs that aim to empower community members while providing education about the consequences of FGM have had an effect on shifting cultural attitudes and beliefs, enabling abandonment of FGM (Diop & Askew, 2009; UNICEF, 2010). Community interventions which focus on allowing members to discuss and debate harmful social norms and traditional practices have shown some evidence of being effective in changing attitudes (Berg & Denison, 2013). Incorporation of multiple strategies at the community level and their evaluation can further knowledge in this area and inform prevention efforts.

An attachment to traditional values with a desire not to abandon ties to cultural roots among migrant communities in HIC was another key propeller of FGM identified in our review, which mirrors prior research (e.g., Mkuwa et al., 2023; Norman et al., 2016). This theme highlights the importance of culture serving as an identity marker to reinforce traditions. However, harmful practices embedded in culture need questioning, and to this end, a Senegalese organization, Tostan, targets female empowerment through education, leadership skills, and development with a precise goal to challenge cultural norms around FGM. Although change may not be immediate, this intervention may have a generational impact (Gruenbaum, 2005). While Tostan’s efforts are commendable, what is unknown is to what extent decision-making around FGM lies with women. A prior study looked at parental decision-making about FGM but explored it in the context of one LIC (Sudan; Sabahelzain et al., 2019). Their study found that mothers had a more decisive role in continuing the practice of FGM, while fathers played a more central role when the decision to abandon the practice was made within families in Sudan. Our review only found parental influence in three studies with one advocating that parents should help abandon the practice (Isman et al., 2013) and the other two noting an equal participation of mother (Strid & Axelsson, 2020) and father (Glover et al., 2017) in the continuation of the practice. Mackie (1996) argues that the practice of FGM in families will continue as long as parents believe that the advantages of FGM outweigh the disadvantages. A prior study by Cappa et al. (2020) found that the risk of FGM decreases if even one parent is opposed to the practice. An understanding in research of how parental attitudes can be shifted to lower the risk of FGM can further inform prevention efforts in HIC.

Persisting with the notion of misconceptions and cultural traditions, two further themes in our review which reflected barriers focused on the maintenance of intergenerational traditions and FGM’s association with hygiene and cleanliness. While prior research supports these findings (e.g., Boyle & Svec, 2019; O’Neill, 2018), interventions do not appear to effectively target these influences (O’Neill, 2018). Straethern (2004) suggests that a woman’s body in many FGM practicing communities is not considered ‘hers’ but is the business of other family and wider community members. Consequently, any misconceived notion of cleanliness is not just about individual hygiene and ‘purity’ but is associated with family members’ standing in the community, with ‘uncut’ females considered dirty and impure which reflects poorly on the family, thus interlinking cleanliness with family honor. Beattie (1991) proposes a bottom-up or participative approach to FGM interventions. This contrasts with top-down and prescriptive intervention approaches identified as prevalent forms of FGM intervention in HIC in a systematic review by Njue et al. (2019). Research by Adinew and Mekete (2017) suggested targeting elder and community influences in anti-FGM interventions in Ethiopia based on the power they uphold in affecting cultural norms and practices. Similarly, a study in Tanzania found older women and community leaders as “key change agents” in fighting against FGM (Mkuwa et al., 2023). Community members’ participatory engagement in co-designing intervening strategies may be an optimal means of both empowering and enabling communities to abandon the practice.

Relatedly, beliefs about social sanctions and a general fear of being shunned by the community represented another theme noted in our review, and this is supported by prior evidence in the field. Omigbodun et al.’s (2022) qualitative study, for instance, not only found stigma around “uncut” females but also a deep-rooted fear of being ostracized from the community, being bullied, and extreme harassment resulting in “social death” among the female participants (p. 12). While this study was conducted with the Izzi community in Southeast Nigeria with only females, the findings are applicable to both male and female migrants to HIC, reflecting the common thread of collectivist attitudes and social interdependency prevalent in FGM practicing communities (Kpanake, 2018). Intervention provisions emphasizing psychological support in the form of counseling for those exposed to social sanctions as well as community-level interventions to ease social stigma need consideration and emphasis by practitioners as potentially optimal means of intervening in HIC.

Similarly, the theme of intergenerational transmission of the practice is related to adhering to cultural roots and traditions and ensuring they are passed down, again emphasizing the socio-cultural influences deeply embedded in communities and reflected in the previously discussed themes. In contrast, the theme of breaking the intergenerational cycle as well as the memory of trauma/pain among FGM victims who had been psychologically and physically harmed by the practice acted as facilitators, motivating women to break the cycle and not subject their own daughters to FGM. There is scarce evidence on what the influencing factors are for women who perpetuate versus those who end the transmission of the practice generationally. More research in this area is needed to inform what may be effective in breaking intergenerational cycles. Further to this, interventions that provide social networking for women and encourage discussions regarding motivations for and against the practice can (a) ease the culture of silence around the topic, (b) allow space for women to discuss issues and exchange ideas, and (c) help empower women to shun harmful familial and communal pressures (Akweongo et al., 2021).

Religious beliefs acted as both a facilitator and barrier to FGM prevention in HCI in our review. Prior research supports religion as a barrier to the abandonment of FGM, especially in LIC. For instance, a study emphasizing religion’s role in practicing FGM in Burkina Faso (West Africa) among Muslims and Christians found that collective interpretation of religious doctrine (even if incorrect) was dominant over any individual interpretation (Hayford & Trinitapoli, 2011). This signifies the role of culture rather than religion in perpetuating FGM. A qualitative study examined religious leaders’ knowledge and influence on FGM among Kurdish communities in Iraq (Ahmed et al., 2018). The study found that religious leaders lacked adequate knowledge about FGM, indicating no health risk from the procedure, and participants from the community were unsupportive of laws to ban FGM, fearing that it may go against their religion. There is scant literature on religion’s facilitative influence on FGM prevention. Our findings had only one study (Isman et al., 2013) in which religious belief acted as a positive influence to abandoning FGM, however, this was acquired through consultation with a religious leader who was knowledgeable about FGM and advised against continuing the practice. Intervention efforts are needed to ensure religious leaders in the communities have adequate knowledge which can help ensure any influence from them on the community is positive and helpful in preventing FGM.

Our systematic review also found that, for some migrant communities, moving to HIC facilitated the discontinuation of FGM with host countries, providing a source of protection with their laws. This was also partly reflected in another theme identified in our review regarding knowledge and awareness. These themes coincide with prior research, which suggests that education on HIC laws, along with actual criminal/court case examples of those who practice FGM, can act as effective deterrents (Berg & Denison, 2013). However, it is also possible that the protective/deterring effects of legislative awareness only readily apply to those already against the practice, but may be too afraid to speak up against it or those who have been victims of FGM. More research uncovering for whom these influences are facilitative can advance knowledge in this area and also inform what can be done to reach those who are not deterred nor protected by these laws.

### Limitations

Our review has a few limitations worth noting. First, our rigid criteria for inclusion resulted in the exclusion of gray literature, which may have added value to the findings. Future systematic reviews should include these sources of information to further knowledge in the field of prevention of FGM. Second, we only included qualitative research and evidence from quantitative research was excluded. This was primarily done to gain a deeper understanding of influences and perspectives around FGM, which are difficult to ascertain from quantitative research. Thirdly, we did not restrict our systematic review to a particular community or culture and while it may be argued that influences may differ across communities, highlighting the common threads and shared motivations is significant and can help inform interventions to create strategies that may benefit a wider population, regardless of origin.

One critical area of investigation that is not discussed in our systematic review, which has implications for the prevention of FGM in HIC, is female genital cosmetic surgery (FGCS) which encompasses several surgical procedures, including labiaplasty (similar to modification of genital tissue that occurs in FGM and carries similar risks; Boddy, 2020). FGCS is mostly elective in nature as medical indications for such procedures are rare (Braun, 2010). In the UK, over 300 girls under the age of 14 underwent FGCS between the years 2006 and 2012 performed by the National Health Service (Liao et al., 2012). Similarly, in the USA, data from the years 2016 to 2019 show that 20% of labiaplasties were performed on those under the age of 18 (Luchristt et al., 2022). While significant, the predicted heterogeneity in study participants, study methods (e.g., quantitative study designs), and findings (e.g., influences on decision making) focused on FCGS was outside the scope of our review and resulted in our decision to not include this literature. However, the legalization of FGCS which has close parallels to FGM, and which is increasingly performed on minors in HIC, presents a clear contradiction and requires further research, especially in investigating its impact on the prevention of FGM in HIC. Despite these limitations, our study provides a valuable contribution to synthesizing and presenting the varied cultural, communal, and traditional influences on FGM in HIC and discusses potential ways that interventions and researchers can help with prevention.

### Implications

Table 6 highlights the key findings of our review and associated implications. Findings from our systematic review can inform efforts to reduce and prevent the harmful practice of FGM in HIC. Elucidating the motivating barriers and facilitators to its prevention enables intervention developers and practitioners to tailor and align intervening strategies to focus on shared influences among communities. The significance of the cultural thread prevalent in many of our identified themes, especially those pertaining to misguided beliefs regarding female virtue and hygiene, beliefs about community sanctions, and a general culture of silence, offers valuable insight. Practitioners need to move away from a focus on the individual or the familial and center their efforts at the community level to shift prevalent and traditional beliefs around FGM. Matanda et al.’s (2023) systematic review of interventions found that community level interventions are effective but “more must be done to innovate with these interventions so that they move beyond affecting attitudes alone to creating behavior change” (p. 1). In particular, involving the community in ways that can help with the prevention of FGM, educating religious leaders of communities, and targeting specific parental influences can help alleviate the negative impact of cultural, religious, and traditional beliefs in perpetuating the practice (Ahmed et al., 2018). The creation of social networks, especially for women, can enhance female empowerment and encourage discussion of FGM in a safe space (Akweongo et al., 2021). Similarly, offering emotional support in the form of therapy and counseling, especially for victims of FGM, can aid the healing process as well as help discontinue intergenerational transmission of FGM.

**Table 6.** Key Findings and Implications.

No.	Findings	Implications
1	A key barrier to the prevention of FGM in HIC includes misguided notions about FGM protecting female virtue.	Intervention strategies to target misguided beliefs on a social and community level can help educate the community and dissociate the practice of FGM and female virtue. More research needs to be conducted to present evidence contrary to the belief, which can be useful in helping to abandon the practice.
2	Attachment to culture and ethnic roots is a key propeller of FGM, and the key drivers of traditional practices stem from the family unit with parents acting as the main decision makers.	Further research to understand parental influences and how to shift parents’ attitudes can lower the risk of FGM and help prevention efforts.
3	Another culturally inherent notion acting as a barrier rested on notions of hygiene and cleanliness associated with FGM.	Stemming from communal influences, this barrier may be targeted by involving influential community members in the co-design and outreach of governmental and intervention efforts to prevent FGM, allowing members to feel empowered to create change.
4	A fear of social sanctions, mirrored in prior research from LIC, was another key barrier to prevention.	Intervention provision can help by providing emotional support to those who want to discontinue the practice but are fearful of the consequences. This may enable them to be steadfast in their decision not to continue FGM. Community-level interventions which focus on the stigma attached to discussing the practice may also help ease the sanctions imposed by community members.
5	Intergenerational transmission of FGM was another barrier found in our review.	Researchers can further knowledge in this area by comparing cycle-breakers to cycle-maintainers and delineating their underlying motivations.
6	A theme of religious beliefs acted as both a barrier and facilitator to FGM prevention in HIC.	More research needs to be conducted on the facilitative influence of religion, which is currently an under-research area. Findings can then be used to counteract the negative and often misinterpreted role of religion in perpetuating FGM in HIC. Targeting religious leaders and educating them about the harmful effects of FGM may also be helpful.
7	A facilitative and protective influence was noted in our review, which centered on HIC legislation against FGM.	Research to better understand for whom this is a deterrent and to identify those populations that are not protected from this influence is needed. This can be useful in guiding legislation and policy to better combat FGM in HIC.

*Note.* FGM = Female Genital Mutilation; HIC = High Income Countries; LIC = Low Income Countries.

### Diversity

Although our review focused on and highlighted FGM in HIC, the population samples of the studies included in the review formed a diverse group of migrant participants who originated from various LMICs. In our review, 14 HIC countries were represented, including Canada, the USA, England, Belgium, and Spain. While not all study authors identified the country of origin of the participants included in their study, for those that did, a total of 12 LMIC countries were represented in the study populations. These were mostly from the sub-Saharan region of Africa, including Sudan, Nigeria, Kenya, and the Ivory Coast as well as East and West African countries, including Ethiopia, Senegal, the Gambia, and Guinea Bissau. There was a slightly higher representation of females than males in the included studies’ populations. The majority of the 14 included studies had both male and female participants (59%), and the remaining 18% had only males, while 23% had only females.

## Conclusion

As an update to a prior systematic review (Berg & Denison, 2013), our study helped identify the various influencing factors that perpetuate or inhibit FGM in HIC. Our study noted barriers to FGM prevention which were primarily rooted in the socio-cultural sphere and appeared common to many FGM practicing communities. Key barriers such as a culture of silence, beliefs about female virtue and hygiene, and social sanctions were identified. Key facilitators which can enable the prevention of FGM were the pain and trauma remembered from having FGM and using this as motivation to discontinue the practice generationally, knowledge of female anatomy, and awareness of legislative protection from host countries. Some influences acted as both facilitators and barriers such as religious beliefs and parental influence. Socio-cultural influences emerge as key motivation, especially for the continuation of the practice. In the context of HIC, interventions need to balance and negotiate migrants’ desire to stay close to their traditions and cultural roots and, at the same time, challenge harmful practices like FGM. Interventions can consider precise influences to develop strategies specifically at the community level to empower, educate, and shift beliefs surrounding FGM.

This systematic review further helps to clarify gaps in knowledge. More research is needed to understand parental influence in families within HIC about continuing or discontinuing FGM. Interventions can use influences (e.g., protective legislation in HIC) that facilitate the abandonment of the practice as leverage to change parental attitudes and ensure a lowered risk of FGM within families in HIC. Research clarifying the differences between women who break the cycle of FGM and those who perpetuate intergenerational transmission of FGM can also provide valuable insight. A detailed exploration of gendered differences in many of the identified beliefs, especially those regarding female virtue and hygiene, can move the field forward, aiding intervention efforts to enable communities to shun the practice of FGM in HIC.

## Supplemental Material

sj-doc-1-tva-10.1177_15248380241229744 – Supplemental material for “All you Gain is Pain and Sorrow”: Facilitators and Barriers to the Prevention of Female Genital Mutilation in High-income Countries Supplemental material, sj-doc-1-tva-10.1177_15248380241229744 for “All you Gain is Pain and Sorrow”: Facilitators and Barriers to the Prevention of Female Genital Mutilation in High-income Countries by Fatima Younas and Leslie Morrison Gutman in Trauma, Violence, & Abuse

sj-docx-2-tva-10.1177_15248380241229744 – Supplemental material for “All you Gain is Pain and Sorrow”: Facilitators and Barriers to the Prevention of Female Genital Mutilation in High-income Countries Supplemental material, sj-docx-2-tva-10.1177_15248380241229744 for “All you Gain is Pain and Sorrow”: Facilitators and Barriers to the Prevention of Female Genital Mutilation in High-income Countries by Fatima Younas and Leslie Morrison Gutman in Trauma, Violence, & Abuse
