# Supplementary material for: “All you Gain is Pain and Sorrow”: Facilitators and Barriers to the Prevention of Female Genital Mutilation in High-income Countries
Source: Trauma Violence Abuse. 2024 Feb 16;25(4):2891–906. doi: 10.1177/15248380241229744 (PMC11370208; doi:10.1177/15248380241229744)
Supplement: sj-docx-2-tva-10.1177_15248380241229744 – Supplemental material for “All you Gain is Pain and Sorrow”: Facilitators and Barriers to the Prevention of Female Genital Mutilation in High-income Countries [file sj-docx-2-tva-10.1177_15248380241229744.docx]

**Quality Appraisal – CASP Qualitative Checklist**

- *Section A: Are the results valid?*
  - Is there a clear statement of aims of research?
  - Are the qualitative methods appropriate?
  - Is the research design appropriate to address aims?
  - Is recruitment strategy appropriate for aims?
  - Has the data been collected appropriately?
  - Is the relationship between researcher and participant considered?
- *Section B: What are the results?*
  - Have ethical issues been considered?
  - Is there a clear statement of findings?
  - Was the data analysis rigorous?
- *Section C: Will the results help locally?*
  - How valuable is the research?
    - What contributions are made to existing knowledge
    - Have new areas of research been identified?
    - Can findings be transferred to other populations?
    - Can the research be used in other ways?
